# Pediatric testicular torsion: does patient transfer affect time to intervention or surgical outcomes at a rural tertiary care center?

**DOI:** 10.1186/s12894-019-0473-5

**Published:** 2019-05-17

**Authors:** Tyler Overholt, Morris Jessop, John Barnard, Osama Al-Omar

**Affiliations:** 1 School of Medicine, West Virginia University, Morgantown, USA; 2 Department of Urology, West Virginia University, Morgantown, USA

**Keywords:** Testicular torsion, Rural, Patient transfer, Orchiectomy

## Abstract

**Background:**

Testicular torsion (TT) is a urologic emergency that requires prompt surgical intervention. In rural Appalachia, patients are often transferred from surrounding communities due to lack of urologic care. We hypothesized that those transferred would have delayed intervention and higher rates of orchiectomy when compared to those who presented directly to our hospital.

**Methods:**

We performed a retrospective review of patient charts with an ICD-9 diagnosis of TT from 2008 to 2016. Patients met inclusion criteria if diagnosis was confirmed by operative exploration. We compared rate of testicular loss and time until surgical intervention between groups.

**Results:**

Twenty-three patients met inclusion criteria (12 transferred, 11 direct). Patient demographics did not significantly differ between groups. Transferred patients had a higher orchiectomy rate (33% v 22%,*p* = 0.41) although this was not statistically significant. Time to surgery from symptom onset was significantly longer in those transferred (12.9 h) compared to those not transferred (6.9 h, *p* = 0.02). Distance of transfer was not correlated with time of delay (r^2^ = 0.063).

**Conclusions:**

Transferred patients with TT have numerically higher rates of orchiectomy which may reach significance in an appropriately powered study, and relative delays in surgical intervention. This study highlights the need for improved access to urologic care in rural areas.

## Background

Testicular torsion (TT) is a urologic emergency where prompt surgical intervention is necessary to prevent testicular loss. TT can occur in male patients at any age, but there is a bimodal distribution which has been described with peaks within the first year of life and between ages 13–16 years old [1]. The etiology of TT can be further classified into two broad categories: intravaginal and extravaginal [2]. Intravaginal TT occurs when the testis twists completely within the tunica vaginalis [3]. This mechanism is the most common etiology and is usually seen in individuals with a bilateral anatomic defect known as the bell clapper deformity [3, 4]. In extravaginal TT, there is a loose attachment between the tunica vaginalis and the scrotal wall early in prenatal development that increases the likelihood of the tunica vaginalis and its contents to twist around the spermatic cord, most commonly in the neonatal period [5, 6].

Management is centered around prompt surgical exploration for every patient with a definitive diagnosis as well as those with inconclusive imaging due to the morbidity associated with delay in management [7]. Delayed intervention can result in permanent ischemic injury of the testis that can lead to long term consequences including testicular loss and infertility [8–10]. Irreversible ischemia from torsion begins in 6 h from symptom onset and the likelihood of testicular salvage is the highest within the first 6 hours [7, 11]. Based on prior literature, treatment with 6 h of symptom onset correlates with 90–100% chance of testicular salvage [12]. However, treatment within 6–12 h decreases salvage rates to 20–50%, and treatment in the 12–24 h range affords only a 0–10% chance of testicular salvage [12]. If surgery is delayed more than 24 h following symptom onset, testicular non-viability is almost inevitable even if the testicular tissue appears viable during surgical exploration [13]. Due to factors such as patient preference, hospital capacity, and access to health care providers capable of managing TT, over 1/3rd of patients with suspected TT are transferred to a tertiary care facility for management, which increases total time of ischemic damage and morbidity [11].

In rural West Virginia, patients often need to be transferred from far distances to seek care from West Virginia University Hospitals due to the lack of Urologic care availability in many areas of the state. This issue is present in rural areas all throughout Appalachia and the rural United States as a whole. Patients with testicular torsion who have to travel from rural areas to facilities capable of managing torsion can be at increased risk for adverse outcomes including orchiectomy. The aim of our study was to look at the relationships between transferring from outside facilities prior to presentation at our facility and the rate of complications and delays in care. We hypothesized that those transferred to our hospital would have a relative delay in surgical intervention and higher rates of orchiectomy compared to those who presented directly to our hospital.

## Methods

After Institutional Review Board approval, a retrospective analysis of patient charts at Ruby Memorial Hospital (RMH) was performed using ICD-9 diagnosis of testicular torsion from 2008 to 2016. For each TT patient, the primary metrics were the surgical outcomes, including rate of orchiectomy between transferred and non-transferred patients, time from symptom onset to presentation, time from symptom onset to surgical intervention, and time from presentation to surgical intervention. We also aimed to characterize laterality of the affected testicle, if imaging was obtained prior to surgical management, and the mean total symptom duration. In TT patients who were transferred from outside facilities, we also aimed to characterize the mode of transfer, the distance of transfer, and relative delay in surgical intervention caused by transfer.

Criteria for inclusion were met if the patient had a diagnosis of testicular torsion that was confirmed by operative exploration. Patients were excluded from the study if suffering from neonatal torsion or if the symptoms were present for over 24 h. Multiple patients were also excluded due to poor documentation of symptom duration.

Patients were separated into those transferred and those who presented directly to our hospital. One hundred and eight patients with testicular torsion were revealed from the ICD-9 search and of those, 23 met inclusion criteria. Twelve patients were transferred from another hospital and sent to RMH due to lack of urologic care at outside facilities and 11 of these patients presented directly to RMH.

Graphpad and Microsoft Excel were utilized for statistical analysis. Normal distribution of data was confirmed using the Shapiro Wilk test. Statistical analyses performed include chi-squared tests for categorical variable data and two-tailed simple t-tests for continuous variable data. Statistical significance was determined by a threshold *p* value < 0.05.

## Results

Mean age of patients was 17.11 ± 5.88 years. 69.6% of patients in the study were Caucasian, 13.0% were Black, 4.4% were Hispanic, and 13.0% were unspecified. 47.8% of patients were affected on the right testicle and 42.2% were affected on the left testicle. Doppler ultrasound was performed in all 23 patients prior to surgical intervention. Mean duration of symptoms for all patients was 10.0 h. Demographic characteristics did not significantly differ between the two patient groups (Table 1).

**Table 1:** Demographic and clinical characteristics of study participants

Characteristics	Patients transferred to RMH	Patients directly presented to RMH
Age	15.12 ± 5.30	19.29 ± 5.92
Ethnicity		
White	11/12 (91.67%)	5/11 (45.45%)
Hispanic	0/12 (0%)	1/11 (9.09%)
Black	0/12 (0%)	3/11 (27.27%)
Unknown	1/12 (8.33%)	2/11 (18.18%)
Mode of transport		
Ambulance	8/12 (72.73%)	
Air	1/12 (8.33%)	N/A
Unknown	3/12 (25%)	
Laterality	5/12 (41.67%) Right	6/11 (54.55%) Right
	7/12 (58.33%) Left	5/11 (45.45%) Left
Doppler US prior to surgical intervention	12/12 (100%)	11/11 (100%)

The mean time to surgical incision from symptom onset in patients who transferred from another facility was 12.9 h. The mean time to surgical incision from symptom onset in patients who presented directly to our tertiary hospital was 6.9 h. Time to surgery from symptom onset was significantly longer in those transferred (*p* = 0.02).

Among transferred patients, 33% underwent orchiectomy on surgical exploration. Of those presenting directly to our hospital, 22% underwent orchiectomy. No statistically significant difference in rate of orchiectomy between the two patient groups was identified (*p* = 0.41).

The mean time to surgical incision from presentation at RMH in patients who transferred from another facility was 2.6 h. The mean time to surgical incision from presentation in those who presented directly to RMH was 3.8 h. Time from presentation to RMH to surgical incision was significantly less in those who transferred than those who presented directly to RMH (*p* = 0.013).

**Fig. 1:**
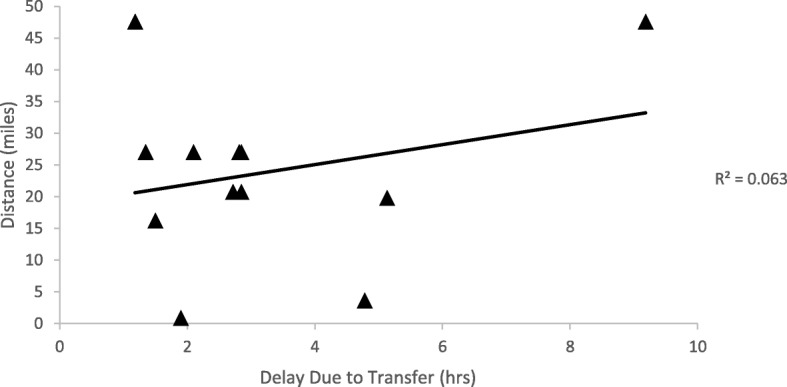
Relationship between delays of care and transfer distances in patients transferred from outside facilities to RMH

Overall, 73% of patients that were transferred to RMH were transferred by ambulance. The mean distance of transfer was 23.8 miles. The mean delay in care from the time of presentation to outside facility to presenting at RMH for all transferred patients was 9.8 h. Distance of transfer was not correlated with the time of delay (r^2^ = 0.063)(Fig. 1).

Approximately 83% of patients who transferred from another facility received follow up care and 91% of patients who presented directly to RMH received follow up care. The mean number of days to receive follow up care in patients who were transferred was 171 days. The mean number of days to receive follow up care in patients who presented directly to RMH was 71 days. There was not a statistically significantly difference in number of follow up days between the two patient groups (*p* = 0.49).

## Discussion

This study focuses on the relationships between patient transfer from rural areas throughout West Virginia to RMH and the clinical and surgical outcomes in patients with testicular torsion. The hypothesis that those transferred to our hospital would have a longer delay in surgical intervention and a higher numerical rate of orchiectomy was supported. There was a statistically significant delay from onset of testicular torsion symptoms to surgical intervention in the patients who were transferred to RMH compared to those who presented to RMH directly. These results suggest that patients who live in rural areas and present to rurally located hospitals first require transfer and travel time that is more likely to result in delays in definitive management. The rate of orchiectomy was 11% higher in patients who transferred to RMH than in the patients who presented to RMH directly. Although this number did not reach statistical significance, most likely due to small sample size of patients, these results suggest that delays in surgical intervention for testicular torsion patients are more likely to be associated with the need for orchiectomy.

Testicular torsion patients who were transferred to RMH had a significant decrease in time from presentation at RMH to surgical incision than patients who presented directly to RMH. There are multiple possible explanations for why these results were seen. One such explanation could be appropriate communication between facilities prior to arrival at RMH to ensure expeditious management. Another explanation may be that diagnostic criteria were completed at the outside hospital, expediting the process at RMH. Other studies have recognized the importance of shorter times from arrival to the operating rooms in patients who have already experienced delays in management due to the need for hospital transfer. One study implemented a standardized track called “Straight to the Operating Room” with a goal of reducing time to surgery, decreased hospitalization costs, and less overall testicular loss [14]. While they did not see a statistically significant reduction in testicular loss, the STOR track did significantly decrease time to surgery and hospitalization cost for affected patients [14]. Additionally, the distance that patients had to travel was not statistically correlated with amount of delay. These results, along with the finding that 73% of patients were transferred via ambulance, suggest that transportation distance itself was not associated with delays in care. Other studies have also shown that travel distance was not statistically correlated with total delay time. One study found that in patients traveling from distances all over 40 miles away, there was no difference in surgical outcomes between patients who travelled by car, ambulance, or helicopter [15].

Patients who transferred to RMH were 8% less likely to receive follow up than those who presented directly to our facility. Although this number did not reach statistical significance, the mean number of days to follow up care was 99.73 days longer in patients who transferred to RMH than patients who presented directly to RMH. These results suggest that geographic proximity to our facility may play a role in receiving follow up care, but due to many factors including post-surgical counseling and communication with patients and their families, a large percentage of the patients are receiving the follow up care that they need.

Fourteen total patients were excluded from this study solely due to symptom duration in excess of 24 h. Of these patients, 11 (78.6%) were transferred from outside facilities and all 14 patients (100%) had an orchiectomy as their surgical outcome. These results suggest the need for community and rural clinician education on recognizing the signs and symptoms of testicular torsion.

## Conclusions

These results imply that patients who live in rural areas may have an increased likelihood to face negative outcomes than patients who live in close enough proximity to a facility with adequate care. This is in conjunction with other studies about delays in care for patients in rural settings. In a study assessing the health status of cancer survivors in rural areas versus urban areas throughout the United States, patients who lived in rural areas were more likely to have poor outcomes including additional comorbidities, worse overall physical and mental health, difficulties with maintaining jobs due to their health [16]. Similar data has been shown for trauma patients where individuals who experience traumas in rural areas have a 50% higher mortality than individuals in urban areas [17]. In a retrospective study looking at prehospital causes of rural fatality rates following motor vehicle crashes in Alabama, it was found that longer emergency medical service response time, longer time on the scene, and longer distances to travel to the scene were all associated with increased mortality in rural trauma patients [18]. Individuals from rural areas face challenges of difficult travel, lack of access to health care facilities, and lack of access to specialty care [19, 20].

This study demonstrated that patients with testicular torsion who transferred from an outside facility prior to presentation at RMH had higher rates of orchiectomy, relative delays in surgical care, less follow up care, and longer times to receive follow up care. This study also highlights the prevalence of delays in diagnosis and presentation, which could be due to gaps in primary care recognition of this urologic emergency.

Delays in management of testicular torsion can result in life long complications and adverse outcomes for individuals affected. In addition to its retrospective design, limitations of the study also include small sample size. However, inclusion and exclusion criteria were formed to be as specific as possible to minimize bias from confounding factors. Future studies may be aimed at looking at testicular torsion patients in rural Appalachia as a whole. This study highlights the need for streamlined access to prompt urologic care in rural areas to minimize the morbidity associated with delay.

## Abbreviations

RMH: Ruby Memorial Hospital
TT: Testicular Torsion
